# Swallowing and choking difficulties as potential markers of FXTAS progression in *FMR1* premutation carriers

**DOI:** 10.1038/s41598-025-25959-5

**Published:** 2025-11-26

**Authors:** Narueporn Likhitweerawong, Federica Alice Maria Montanaro, Ellery R. Santos, Jun Yi Wang, Waralee Dejputtawat, Udayakumar Narasimhan, Jessica Klusek, Nell Maltman, Andrea Schneider, Flora Tassone, Randi J. Hagerman

**Affiliations:** 1 University of California Davis MIND Institute, UC Davis Health, Sacramento, CA 95817 USA; 2 Department of Pediatrics, Faculty of Medicine, Chiang Mai University, Chiang Mai, 50200 Thailand; 3 Center of Clinical Epidemiology and Clinical Statistic, Faculty of Medicine, Chiang Mai University, Chiang Mai, 50200 Thailand; 4 Department of Education, Psychology, Communication, University of Bari Aldo Moro, 70122 Bari, Italy; 5 Center for Neurodegenerative Diseases and the Aging Brain, Department of Clinical Research in Neurology, University of Bari ’Aldo Moro’, 73039 Tricase, Italy; 6 Department of Pediatrics, School of Medicine, University of California Davis, Sacramento, CA 95817 USA; 7 Center for Mind and Brain, University of California Davis, Davis, CA 95618 USA; 8 Department of Pediatrics, Nakornping Hospital, Chiang Mai, 50180 Thailand; 9 Karthikeyan Child Development Unit, Department of Pediatrics, SRIHER, Porur, 600116 India; 10 Communication Sciences and Disorders, Arnold School of Public Health, University of South Carolina, Columbia, SC 29208 USA; 11 Department of Speech, Language, and Hearing Sciences, University of Arizona, Tucson, AZ 85721 USA; 12 Department of Biochemistry and Molecular Medicine, School of Medicine, University of California Davis, Sacramento, CA 95817 USA

**Keywords:** Fragile x premutation, *FMR1* premutation, Dysphagia, Swallowing problems, Fragile x-associated Tremor/Ataxia syndrome, FXTAS, Diseases, Medical research, Neurology, Neuroscience

## Abstract

Fragile X-associated tremor/ataxia syndrome (FXTAS) affects motor and coordination pathways and is linked to swallowing and choking difficulties, which can lead to aspiration pneumonia, a leading cause of death in late-stage FXTAS. Despite their severity, these issues are under-investigated. This study examined their association with FXTAS stages and potential as markers of disease progression in ***FMR1*** premutation (PM) carriers. A secondary analysis of Genotype-Phenotype cohort data (2017–2025, MIND Institute, UC Davis) examined swallowing/choking problems, FXTAS stage, neuroimaging, and psychological distress (Symptom Checklist-90-Revised; SCL-90-R). Associations between independent and dependent variables were tested using Generalized Estimating Equation (GEE) regression due to their correlated data. The study included 169 PM carriers (mean age 65 ± 10.9 years; 54% male), with approximately 35% reporting swallowing/choking difficulties. After adjusting for age and sex, individuals in the severe stage of FXTAS (stage 4–5) had a significantly higher risk of swallowing/choking problems compared to those without FXTAS (adjusted odds ratio [aOR] = 4.17; 95%CI = 1.28–13.58). PM carriers with swallowing/choking problems showed a significantly increased association with magnetic resonance imaging (MRI) findings of moderate to severe abnormalities in several brain regions, including cerebral atrophy (aOR = 2.69, ***p*** = 0.027), cerebellar atrophy (aOR = 3.34, ***p*** = 0.013), cerebellar white matter hyperintensity (aOR = 3.33, ***p*** = 0.012), and pons white matter hyperintensity (aOR = 3.93, ***p*** = 0.035). Swallowing/choking problems are common in FXTAS, particularly in later stages, and may represent an important clinical marker of disease progression. These patients should be referred to speech-language pathologists for evaluation and treatment. Such interventions could reduce morbidity-mortality associated with these problems.

**Supplementary Information:**

The online version contains supplementary material available at 10.1038/s41598-025-25959-5.

## Introduction

The *FMR1* premutation (PM) is characterized by an expansion of 55 to 200 cytosine-guanine-guanine (CGG) trinucleotide repeats within the *FMR1* gene, situated on the X chromosome. This mutation represents an intermediate state between the normal range (up to 54 repeats) and the full mutation (> 200 repeats) observed in Fragile X Syndrome (FXS)^1^. Individuals with the PM typically produce slightly low, normal, or even elevated levels of Fragile X Messenger Ribonucleoprotein (FMRP)^2^, but they are not immune to clinical manifestations. While they do not exhibit the profound cognitive deficits characteristic of FXS, people with PM are at an elevated risk for a range of clinical conditions, some of which are severe and progressive. These conditions, that together take the name of Fragile X Premutation associated Conditions (FXPAC)^3^ include: Fragile X-associated Tremor/Ataxia Syndrome (FXTAS), a late-onset neurodegenerative disorder predominantly affecting males, but affecting also females; Fragile X-associated Primary Ovarian Insufficiency (FXPOI), which is linked to premature ovarian failure before age 40 in females; and Fragile X-associated Neuropsychiatric Disorders (FXAND) that reflect a spectrum of psychiatric and neurological symptoms, including anxiety, depression, attention-deficit/hyperactivity disorder (ADHD), and insomnia^4^. For the purpose of the present study, we will focus on one of these conditions – FXTAS.

Diagnosis of FXTAS typically involves a combination of clinical features of action tremor and balance problems, molecular testing to identify PM status, and neuroimaging to detect characteristic brain changes of white matter hyperintensities such as the middle cerebellar peduncle (MCP) sign and/or splenium sign^5,6^. However, there is less understanding of the longitudinal progression of the condition, which limits our understanding of how symptoms may develop and change over time. Prior to a FXTAS diagnosis, many individuals exhibit preFXTAS symptoms during the ages of 50 to 70 that can be different between sexes, and which may include intermittent mild tremors, memory and executive function (EF) deficits, anxiety, and mood disturbances^7–9^. These signs may serve as critical indicators for early intervention, so full characterization of the preFXTAS phenotype is essential.

From a pathological perspective, it has been shown that PM carriers with FXTAS have intranuclear inclusions in both astrocytes and neurons in the brain, potentially leading to white matter disease that disrupts multiple brain functions, particularly those related to motor and coordination control^10^. Swallowing and choking difficulties—clinically referred to as dysphagia – are often attributed to motor and coordination impairments. Dysphagia is estimated to affect about 1 in 25 adults^11^ with prevalence increasing with age. Older adults with neurodegenerative disorders are at particularly high risk for dysphagia. For example, approximately 50% to 80% of individuals with Parkinson’s disease experience swallowing difficulties^12,13^, and the prevalence ranges from 84% to 93% in those with Alzheimer’s disease^14^. These swallowing and choking problems can result in serious complications, such as aspiration pneumonia which is a prevalent cause of death each year in the United States that accounts for a substantial proportion of pneumonia-related hospitalizations and mortality, especially among older adults and those with neurological conditions^15^.

Moreover, individuals with swallowing problems are more likely to have mental health comorbidities, as it can have a substantial impact on quality of life, namely by limiting shared meals and related social engagements^16^. Although swallowing and choking difficulties are commonly considered through the lens of motor and coordination dysfunction, these symptoms can also associate with psychological distress, as evidenced by strong positive correlation between dysphagia and depression and anxiety reported in other neurodegenerative diseases^17,18^. Given that the preFXTAS and FXTAS phenotypes include neuropsychiatric difficulties^7^, it is possible that swallowing and choking problems in PM carriers may present alongside mental health phenotypes such as anxiety and depression.

Despite their clinical importance, swallowing and choking difficulties in FXTAS have not been thoroughly investigated. There remains a limited understanding of how these symptoms evolve across different stages of FXTAS or whether they correlate with magnetic resonance imaging (MRI)-detected brain abnormalities. Addressing these gaps of knowledge could significantly enhance clinical assessment and monitoring of disease progression. This study aimed to evaluate whether there is an association between swallowing/choking problems and FXTAS in PM carriers and whether swallowing/choking problem could be a possible marker of severe stages of FXTAS.

## Results

### Descriptive statistics

A total of 169 PM carriers with 226 observations were included in the analysis. The mean age of participants at the first visit was 65.0 years (*SD* = 10.9), and 54% were male. Over two-thirds (69%) of participants had low PM alleles (cytosine-guanine-guanine (CGG) repeats 55–99), and the remaining 31% had high PM alleles (CGG repeats 100–200). Based on FXTAS staging, the distribution across clinical stages at the first visit was as follows: 19% of participants had no signs of FXTAS (stage 0), 8% were at stage 1, 18% at stage 2, 40% at stage 3, and 15% were classified as stage 4–5. There were no patients with FXTAS stage 6 (bed-ridden) in the sample given that individuals at this stage are unable to participate in clinic assessment. Swallowing and choking difficulties were reported in approximately 35% of the PM carriers. The proportion of individuals reporting these issues in each stage of FXTAS was: no FXTAS (8 of 29 individuals; 28%), stage 1 (4 of 12; 33%), stage 2 (5 of 28; 18%), stage 3 (22 of 62; 35%), and stages 4–5 (17 of 24; 71%). Participants were categorized into subgroups according to endorsement/non-endorsement of swallowing/choking problems. Comparisons of demographic and clinical characteristics between these subgroups are summarized in Table 1. There were no statistically significant differences in age, sex, or PM CGG repeat category (i.e., “low” vs. “high”) between individuals with and without swallowing/choking problems.

**Table 1:** Comparisons of demographic and clinical characteristics at the first time point of visit between PM carriers with and without swallowing/choking problems.

Variables	Swallowing/choking problems endorsed (*n* = 59)	Swallowing/choking problems not endorsed (*n* = 110)	*P*-Value	Missing data, *n* (%)
Sex, *n* (%)				
Female	28 (47%)	49 (45%)	0.717	0
Male	31 (53%)	61 (55%)		
Age, mean (SD)	66.51 (8.91)	64.48 (11.76)	0.249	0
Race, *n* (%)				
White	56 (95%)	98 (91%)	0.435	3 (2%)
African-American	-	3 (3%)		
Asian	2 (3%)	4 (4%)		
Native Hawaiian or other Pacific Islander	1 (2%)	-		
Others	-	2 (2%)		
Ethnic, *n* (%)				
Non-Hispanic or Latino	44 (90%)	81 (93%)	0.525	33 (20%)
Hispanic or Latino	5 (10%)	6 (7%)		
Premutation, *n* (%)				
Low (CGG repeats 55–99)	38 (68%)	71 (70%)	0.858	11 (7%)
High (CGG repeats 100–200)	18 (32%)	31 (30%)		
FXTAS Stage at the 1st visit, *n* (%)				
0	8 (14%)	21 (21%)	0.002	14 (8%)
1	4 (7%)	8 (8%)		
2	5 (9%)	23 (23%)		
3	22 (39%)	40 (40%)		
4–5	17 (30%)	7 (7%)		

### Association between FXTAS and swallowing/choking problems

A statistically significant association was observed between FXTAS stage and the presence of swallowing/choking difficulties. After controlling for age and sex, the severe stage of FXTAS (stage 4–5) was associated with significantly increased the risk of having swallowing/choking problems (adjusted odds ratio [aOR], 4.17; 95% CI, 1.28–13.58) compared to those without FXTAS (Table 2). When including the interaction term between time (visit) and FXTAS stage in the model, the association between late stage FXTAS and swallowing/choking problems remained consistent (aOR, 3.92; 95% CI, 1.09–14.09). However, there was no significant interaction effect, indicating that the increased risk of swallowing or choking problems did not vary significantly over time by FXTAS stage (Table S1).

**Table 2:** Generalized estimating equation regression (GEE) testing the effect of FXTAS stage on swallowing/choking problems (*n* = 165, number of observations = 226).

Variables	Adjusted Odds Ratio	SE	95% CI	*P*-Value
FXTAS Stage (Reference = FXTAS Stage 0 or no FXTAS symptoms)				
1	1.33	0.93	0.33–5.28	0.686
2	0.75	0.46	0.23–2.47	0.640
3	1.70	0.90	0.60–4.79	0.319
4–5	4.17	2.51	1.28–13.58	0.018

Adjusted GEE model controlled for age and sex and unstructured correlation pattern within same patients.

### Association between MRI findings and swallowing/choking problems

After adjusting for age and sex, swallowing/choking problems were significantly associated with advanced brain MRI findings (moderate-to-severe severity grading) in several brain areas. The brain features significantly affected include cerebral atrophy, cerebellar atrophy, cerebellar white matter hyperintensity, and pons white matter hyperintensity (Table 3). However, when the interaction term between time (visit) and swallowing/choking problems was included in the model, the interaction effect was not statistically significant (Table S2).

**Table 3:** Generalized estimating equation regression (GEE) of swallowing/choking problems on MRI findings (*n* = 116, number of observations = 133).

MRI findings	Adjusted Odds Ratio	SE	95% CI	*P*-Value
Cerebral atrophy	2.69	1.20	1.16–6.47	0.027
Cerebellar atrophy	3.34	1.63	1.29–8.67	0.013
Cerebral white matter hyperintensity	1.22	0.47	0.57–2.60	0.611
Cerebellar white matter hyperintensity	3.33	1.60	1.30–8.53	0.012
Middle cerebellar peduncle white matter hyperintensity	1.39	0.51	0.67–2.86	0.374
Pons white matter hyperintensity	3.93	2.55	1.10–14.03	0.035
Sub-insular white matter hyperintensity	1.07	0.50	0.43–2.69	0.881
Periventricular white matter hyperintensity	1.93	0.90	0.77–4.79	0.159
Splenium of corpus collosum white matter hyperintensity	1.39	0.56	0.63–3.08	0.412
Genu of corpus callosum white matter hyperintensity	1.92	0.79	0.86–4.30	0.112
Corpus callosum thinning	0.71	0.20	0.42–1.22	0.222

Adjusted GEE model controlled for age and sex and unstructured correlation pattern within same patients.

### Association between swallowing/choking problems and psychological distress

To examine whether increased psychological distress is associated with more frequent reports of swallowing or choking difficulties, GEE regression models, adjusted for age and sex, were performed separately for each symptom subdomain of the SCL-90-R assessment. The results revealed significant associations between the overall scores of the SCL-90-R (i.e., GSI, PSDI, and PST) and swallowing/choking problems. When examining the subscales of the SCL-90-R, significant associations were also found between certain subscales (i.e., anxiety and psychoticism) and swallowing/choking problems. PM carriers with higher scores on these subscales were more likely to report swallowing or choking difficulties. In contrast, other subscales of the SCL-90-R did not show statistically significant associations (Table 4).

**Table 4:** Generalized estimating equation regression (GEE) of SCL-90-R subscales on swallowing/choking problems (*n* = 137, number of observations = 175).

SCL-90-*R* subscales T scores	Adjusted Odds Ratio	SE	95% CI	*P*- value
Somatization	1.02	0.01	1.00–1.05	0.100
Obsessive-compulsive	0.99	0.01	0.97–1.00	0.106
Interpersonal sensitivity	1.01	0.02	0.98–1.04	0.464
Depression	1.03	0.02	1.00–1.07	0.055
Anxiety	1.04	0.02	1.001–1.07	0.040
Hostility	1.02	0.02	0.99–1.07	0.127
Phobic anxiety	1.03	0.02	0.99–1.06	0.156
Paranoid ideation	1.03	0.02	0.99–1.06	0.101
Psychoticism	1.04	0.02	1.01–1.08	0.019
Global severity index	1.04	0.02	1.01–1.08	0.014
Positive symptom distress index	1.03	0.02	1.002–1.07	0.034
Positive symptom total	1.04	0.02	1.01–1.08	0.026

Adjusted GEE model of SCL-90-R subscales on swallowing/choking problems were separately operated and controlled for age and sex and unstructured correlation pattern within same patients.

## Discussion

This study found that about 35% of PM carriers aged 16–92 experience symptoms of swallowing/choking problems, with these symptoms seen in carriers with and without FXTAS. However, the proportion of PM carriers reporting swallowing/choking problems increased with the progression of FXTAS stage, especially in the late stage of FXTAS (stage 4–5) when as many as 70% of FXTAS patients endorsed problems with swallowing/choking. After controlling for age and sex, individuals in the severe stage of FXTAS (stage 4–5) had a significantly higher risk of swallowing/choking problems compared to those without FXTAS. Additionally, our findings suggest that swallowing/choking issues might be a marker for severe progression of disease, as swallowing/choking problems were more common among FXTAS patients with moderate-to-severe MRI findings compared to FXTAS patients with none-to-mild MRI findings, including cerebral atrophy, cerebellar atrophy, cerebellar white matter hyperintensity, and pons white matter hyperintensity. PM carriers with higher psychological distress scores in anxiety and psychoticism were more likely to experience swallowing/choking problems.

In our cohort, swallowing and choking problems were particularly frequent in late-stage FXTAS (stages 4–5), when severe motor impairment often necessitates an assistive device such as a cane, walker, or wheelchair. For comparison, the estimated prevalence of swallowing/choking problems in the general community-dwelling elderly population over the age of 50 is approximately 15–22%^19,20^. While no prior studies have specifically investigated the prevalence of swallowing and choking difficulties in PM carriers, these issues are prevalent in other neurodegenerative disorders such as Parkinson’s disease, amyotrophic lateral sclerosis (ALS), and Alzheimer’s disease, with reported rates around 80–90% during the courses of diseases^21^. The high proportion of PM carriers reporting swallowing/choking problems, especially in later stages of FXTAS observed in this study, are consistent with these previous reports. Our findings highlight swallowing/choking difficulties as a very common clinical feature of FXTAS, underscoring the need for a proactive, multidisciplinary approach to detect symptoms early in the FXTAS disease course and initiate interventions to prolong swallowing function into the neurodegenerative disease course and improve quality of life.

We found that swallowing/choking problems are associated with cerebral atrophy, cerebellar atrophy, cerebellar white matter hyperintensity, and pons white matter hyperintensity. These findings suggest that swallowing/choking-related symptoms may reflect underlying neurodegenerative processes that are more prominent in the later stages of FXTAS. To further explore whether these associations change over time, an interaction term between time (i.e., study visit) and the presence of swallowing/choking problems was added to the regression model. However, the interaction effect was not statistically significant. This suggests that the observed associations likely reflect a more persistent relationship with disease severity, rather than a progressive change driven by time. However, other MRI findings such as cerebral white matter hyperintensity, MCP white matter hyperintensity, periventricular white matter hyperintensity, sub-insular white matter hyperintensity, splenium of corpus collosum white matter hyperintensity, genu of corpus callosum white matter hyperintensity, and corpus callosum thinning did not show significant associations with swallowing/choking problems. Swallowing involves complex motor and coordination functions. Swallowing difficulties are believed to stem from neurodegenerative changes in brain regions responsible for coordinating swallowing, such as the brainstem and associated cranial nerves^22^, which is occasionally reported as a clinical manifestation in FXTAS^23,24^. Some radiological features that are considered hallmarks of FXTAS, such as the MCP sign^25–27^ were not observed as significantly associated with swallowing/choking problems, although it is notable that our FXTAS sample included roughly equal representation of males and females and the MCP sign is far less common in females with FXTAS^25^. The cerebellum, the brain region initially affected in FXTAS and also involved in swallowing function^28–30^, did show an association with swallowing/choking problems. While the severity of FXTAS progression is often evaluated using clinical indicators such as tremor, gait instability, balance issues, and their impact on daily functioning, neuroimaging findings can provide valuable support for the clinical diagnosis and progression of FXTAS^31,32^. In our study, swallowing difficulties were associated with both more severe grades of brain pathology and FXTAS stage, suggesting that the presence of swallowing issues, even in the absence of severe ataxia, may serve as a critical warning sign of advancing neurodegeneration, emphasizing the need for vigilant monitoring and early intervention.

There is a high prevalence of psychological distress among PM carriers, with the severity of psychological symptoms related to both genetic factors such as CGG repeat length^33,34^, and to environmental influences such as the burdens of caregiving responsibilities and concerns about their own physical health^35–37^. We explored the potential association between swallowing difficulties and psychological distress using the SCL-90-R. Our analysis revealed trends suggesting associations between swallowing/choking symptoms and specific domains of psychological distress, which is consistent with previous study^38^. We found that PM carriers with higher psychological distress scores in the anxiety and psychoticism subdomains were more likely to report swallowing and choking problems. However, the causal relationship between these variables is unclear; swallowing/choking problems can be a source of distress leading to elevated anxiety, but patients with anxiety may also experience heightened sensitivity to swallowing difficulties, amplifying somatic complaints^39^, and bidirectional effects are likely^17^. Psychological distress may emerge in the early stages of FXTAS, as PM carriers become more aware of developing motor and coordination deficits, which can lead to increased distress and anxiety^40,41^. Ultimately, psychological distress may create a vicious cycle by exacerbating physical symptoms such as globus sensation and dysphagia^42^. This reflects the bidirectional relationship between dysphagia-related symptoms and psychological distress, in which each condition reinforces the other^17^.

To the best of our knowledge, this is the first study to evaluate swallowing problems across the stages of FXTAS, highlighting how common these issues are among PM carriers, particularly in the later stages of the disorder. Individuals who do not currently report swallowing or choking problems should still be closely monitored, as these symptoms may emerge later in the course of disease. Timely management is key to reducing complications, morbidity, and mortality associated with swallowing/choking difficulties. Patients should be referred to speech-language pathology for swallowing evaluation and treatment, and to gastroenterology or other specialties as needed for further work-up. Psychotherapy is recommended for psychoeducation, emotional support, and to improve adherence to physical and behavioral interventions. Effective dysphagia intervention not only enhances swallow function and eating outcomes, but can also yield improved psychological health and quality of life, with stronger outcomes when treatment is initiated early in the course of neurodegenerative disease^43–45^.

This study has several limitations. First, the sample size of participants in the later, more severe stages of FXTAS (stages 4–5) was relatively small. Second, although the interaction effect of time in the relationship between swallowing/choking problems and MRI abnormalities was not statistically significant, it is important to note that this result may be influenced by the duration of patient follow-up. Longer follow-up periods may be necessary to detect more robust interaction effects or to capture the progression of clinical and neuroimaging features over time. Since males are more commonly and more severely affected than females in FXTAS, there may be interaction effects on the associations mentioned earlier. Future studies investigating sex as a potential modifier of these associations would indeed help strengthen and more comprehensively interpret the findings. Third, while dysphagia is primarily mediated by the bulbar centers in the brainstem, as well as by cerebellar and cranial nerve pathways, brainstem (bulbar) imaging data were not collected in our cohort. The absence of these data may limit the interpretation of the overall relationship between neuroanatomical changes and swallowing/choking difficulties. Fourth, potential confounding factors, such as comorbid conditions (e.g., stroke, traumatic brain injury) or the use of medications, were not collected, and these may have contributed to the observed swallowing difficulties. Lastly, self-report questionnaires may limit certain interpretations. The self-administered SCL-90 scale represents a limitation when assessing possible psychotic symptoms, as it may not fully capture clinically significant psychosis in this population. Swallowing/choking problems were measured using a self-report binary (i.e., presence/absence of problems). This method alone cannot adequately capture the full scope of swallowing/choking difficulties, and its associated stages (e.g., oral, pharyngeal, esophageal). Identifying the exact origin of swallowing and choking problems in FXTAS is challenging, as both central and peripheral nervous system impairments can be involved. For instance, esophageal neuropathy, a peripheral nervous system issue, can contribute to swallowing difficulties. Additionally, the underlying mechanisms of these symptoms, whether due to anatomical abnormalities or functional impairments, were not assessed in this study. Future research incorporating such evaluations could provide a more comprehensive understanding of swallowing and choking problems in this population and allow for more personalized management strategies based on the specific underlying causes.

In conclusion, swallowing and choking problems are common in FXTAS, particularly during the more advanced stages of the disease. The current findings highlight the importance of recognizing swallowing/choking problems not only as a complication, but as potential manifestations of the neurodegenerative process during FXTAS progression. Symptom monitoring and prompt referral for formal swallowing assessment and initiation of treatment is critical to reduce the risk of aspiration, malnutrition, and respiratory complications, thereby potentially decreasing the morbidity and mortality associated with the progression to severe FXTAS. Future studies should focus on expanding cohort sizes, including larger and more diverse sample of both female and male premutation carriers in order to ensure greater representation across all stages of FXTAS and to better understand potential sex- and stage-related differences.

## Methods

### Study design and participants

This study is a secondary analysis utilizing data from the Genotype-Phenotype longitudinal studies conducted at the UC Davis MIND Institute from 2017 to 2025. The study protocol enrolled male and female participants between the ages of 8 and 85 years, both with and without PM. Recruitment strategies included in-person solicitation, telephone outreach, and public advertisement. The study protocol consisted of 2–3 days of testing and included a comprehensive medical history, neuropsychological and behavioral assessments, neurological examination, balance and tremor evaluation, MRI imaging, venipuncture for blood sample collection, and a small skin biopsy. Participants were recruited primarily through the Fragile X Research and Treatment Center at the UC Davis MIND Institute. Recruitment sources included established clinical and research program contacts, as well as new families seeking clinical services or information about ongoing research. Interested individuals and families were invited to contact the study coordinator directly for participation. Inclusion criteria were identical for males and females and included the confirmed fragile X premutation via genetic testing (FMR1 CGG repeat expansion between 55 and 200), and absence of any life-threatening illness other than FXTAS. Participants in the study were asked to return for follow-up assessments every two years for a total of up to three visits over a five-year period. The study was conducted according to the guidelines of the Declaration of Helsinki and approved by the Institutional Review Board of the University of California Davis School of Medicine. Informed consent was obtained from all subjects involved in the study. An Institutional Review Board-approved informed consent to have their medical histories reviewed and their cases published for medical and scientific purposes.

### Measures

#### Demographics and swallowing/choking problems

Baseline characteristics such as age, sex, race, ethnic were collected. Participants underwent a medical history review and a physical/neurological examination performed by an expert physician (R.J.H.) with extensive experience in fragile X-associated conditions. History included information on neurological and physical health including swallowing/choking problems. Swallowing and choking problems were assessed by asking whether participants currently had difficulties in swallowing while eating solid or liquid foods, feeling of food getting stuck, or coughing or choking while eating. Response was recorded in a binary checkbox format indicating the presence/absence of these problems, using a questionnaire developed for the Genotype-Phenotype study cohort. Neurological issues, including tremors, coordination, muscle tone, reflexes, strength, sensation, and gait, were covered in depth during both the history and physical/neurological examinations. Diagnosis and staging of FXTAS were performed and staged 0–6, according to functional impairment: stage 0: no FXTAS symptoms; stage 1: a subtle or questionable gait instability and/or tremor; stage 2: a minor but clearly detectable balance problem and/or tremor with minimal interference in daily activities; stage 3: moderate tremor or balance problems that significantly interferes with daily activities and reporting occasional falls; stage 4: severe tremor or balance problems, needing a cane or walker; stage 5: daily use of a wheelchair for mobilization; and stage 6: bedridden^40^.

#### Neuroimaging

Standard structural MRIs, including T1-weighted, T2-weighted, and fluid-attenuated inversion recovery (FLAIR) scans were acquired for each study participant using a 3T Siemens TIM Trio (2007–2023) and Prisma (2023–2025) MRI System available at the Imaging Research Center—University of California Davis Health. More details of the MRI acquisition protocol can be found in Hessl et al. (2025)^46^. MRI changes were rated as none, mild, moderate, and severe of the MCP sign, hyperintensities in the pons/sub-insular/periventricular/splenium, cerebral/cerebellar atrophy, cerebral/cerebellar white matter hyperintensity, and rated as whether there was thinning of the corpus callosum and hyperintensity in genu of the corpus callosum.

#### Psychological distress

Self-administered psychological assessment, the Symptom Checklist-90-Revised (SCL-90-R), was performed to assess subjective psychopathology and evaluated by licensed psychologists. This tool measures 9 symptom dimensions which are somatization, obsessive-compulsive behavior, interpersonal sensitivity, depression, anxiety, hostility, phobic anxiety, paranoid ideation, psychoticism and included three global indices: the global severity index (GSI) for overall psychological distress, the positive symptom distress index (PSDI) for intensity of symptoms, and the positive symptom total (PST). Higher T-scores reflect higher psychological distress. It has adequate reliability and validity^47^ and has been used widely in previous premutation study^38^.

#### FMR1 DNA testing

Molecular genetic analysis included the CGG repeat number and methylation status. *FMR1* specific polymerase chain reaction assays and Southern blot analyses were carried out for all participants from peripheral blood as previously described^48,49^. Only participant carriers of PM (55–200 CGG repeats) were included in this study. PM range was determined by the number of CGG repeats, with low PM defined as 55–99 repeats and high PM as 100–200 repeats^50^.

#### Statistical analysis

The statistical analyses were conducted using Stata version 16 (StataCorp, College Station, Texas, USA)^51^. The descriptive data was presented as number (percentage) for categorical data and mean (SD) for normally distributed continuous data. Chi-square and student t-test were performed to investigate the differences of the baseline characteristics such as sex, age, and premutation status (low vs. high CGG repeats) between the two groups of PM with and without swallowing/choking problems, based on types of variables. Since the cohort included participants who had both single and multiple visits, to assess the association between FXTAS and swallowing/choking problems, we conducted Generalized estimating equation (GEE) regression to account for the correlation of repeated measures within individuals. A GEE model was fitted with a binomial distribution and logit link function, swallowing/choking problems (binary variable) as the dependent outcome and FXTAS stage (stage 0,1,2,3, and 4–5) as a predictor. FXTAS stages 4 and 5 were collapsed due to small sample sizes and their shared advanced motor impairment. We specified an unstructured working correlation structure to model within-subject correlations across multiple visits. The final model was adjusted for age and sex. Results were reported as odds ratios with 95% confident intervals (95% CI). To identify potential markers of moderate to severe brain MRI findings in individuals with FXTAS, GEE regression models were conducted, with swallowing or choking problems as the predictor and moderate-to-severe grade MRI findings as the binary outcome (non-to-mild vs. moderate-to-severe grading severity rating). All models examined the associations between these variables, adjusting for age, sex, and considering for the interaction terms between time (visit) and the predictor. Additionally, to assess whether increased psychological distress is associated with more reported swallowing or choking problems, a separate GEE model was used with each SCL-90-R T-score subscale as the predictor and swallowing or choking problems as the outcome variable. A p-value of less than 0.05 was considered statistically significant.

## Supplementary Information

Below is the link to the electronic supplementary material.

Supplementary Material 1

## Data Availability

The datasets generated during and/or analysed during the current study are available from the corresponding author on reasonable request.
