# Supplementary material for: Swallowing and choking difficulties as potential markers of FXTAS progression in FMR1 premutation carriers
Source: Sci Rep. 2025 Nov 26;15:42164. doi: 10.1038/s41598-025-25959-5 (PMC12657983; doi:10.1038/s41598-025-25959-5)
Supplement: Supplementary file 1 — Supplementary Material 1 [file 41598_2025_25959_MOESM1_ESM.pdf]

Supplementary File

Table S1. Generalized estimating equation regression (GEE) of stage of FXTAS on swallowing/choking problems (*n* = 165, number of observations = 226)

**Variables**	**Model with interaction term**			
	**Adjusted Odds Ratio**	**SE**	**95% CI**	**P-Value**
FXTAS Stage (Reference = FXTAS Stage 0 or no FXTAS symptoms)				
1	1.32	0.93	0.33 – 5.25	0.690
2	0.74	0.45	0.22 – 2.43	0.619
3	1.65	0.89	0.57 – 4.73	0.353
4-5	3.92	2.56	1.09 – 14.09	0.037*
FXTAS stage x Time (visit) interaction	1.01	0.05	0.93 – 1.10	0.812

Note. Adjusted GEE model controlled for age and sex and unstructured correlation pattern within same patients. *p <0.050

Table S2. Generalized estimating equation regression (GEE) of swallowing/choking problems on MRI findings (*n* = 116, number of observations = 133)

**MRI findings**	**Model with interaction term**			
	**Adjusted Odds Ratio**	**SE**	**95% CI**	**P-Value**
Cerebral atrophy				
Swallowing/choking problems	4.33	3.74	0.80 – 23.56	0.090
Swallowing/choking problems x Time (visit) interaction	0.71	0.37	0.25 – 1.97	0.507
Cerebellar atrophy				
Swallowing/choking problems	3.41	3.16	0.56 – 20.91	0.184
Swallowing/choking problems x Time (visit) interaction	0.96	0.53	0.35 – 2.80	0.979
Cerebral WM hyperintensity				
Swallowing/choking problems	0.89	0.64	0.21 – 3.68	0.870
Swallowing/choking problems x Time (visit) interaction	1.26	0.52	0.56 – 2.84	0.870
Cerebellar WM hyperintensity				
Swallowing/choking problems	10.47	12.24	1.06 – 103.47	0.040*
Swallowing/choking problems x Time (visit) interaction	0.54	0.41	0.12 – 2.44	0.420

Middle cerebellar peduncle white matter hyperintensity				
Swallowing/choking problems	3.65	3.05	0.71 – 18.73	0.120
Swallowing/choking problems x Time (visit) interaction	0.61	0.26	0.27 – 1.42	0.253
Pons white matter hyperintensity				
Swallowing/choking problems	16.30	20.94	1.31 – 202.18	0.030*
Swallowing/choking problems x Time (visit) interaction	0.33	0.31	0.05 – 2.09	0.283
Sub-insular white matter hyperintensity				
Swallowing/choking problems	0.71	0.58	0.14 – 3.58	0.675
Swallowing/choking problems x Time (visit) interaction	1.37	0.67	0.52 – 3.59	0.524
Periventricular white matter hyperintensity				
Swallowing/choking problems	0.61	0.49	0.12 – 3.00	0.544
Swallowing/choking problems x Time (visit) interaction	2.42	1.21	0.90 – 6.47	0.079
Splenium of corpus collosum white matter hyperintensity				
Swallowing/choking problems	1.13	0.90	0.24 – 5.37	0.874
Swallowing/choking problems x Time (visit) interaction	1.16	0.56	0.45 – 2.99	0.758
Genu of corpus callosum white matter hyperintensity				
Swallowing/choking problems	0.44	0.41	0.07 – 2.79	0.382
Swallowing/choking problems x Time (visit) interaction	3.69	2.52	0.96 – 14.10	0.057
Corpus callosum thinning				
Swallowing/choking problems	0.88	0.46	0.31 – 2.48	0.802
Swallowing/choking problems x Time (visit) interaction	1.00	0.29	0.57 – 1.76	0.999

Note. Adjusted GEE model controlled for age and sex and unstructured correlation pattern within same patients. **p* <0.050
